# An optimized culturomics strategy for isolation of human milk microbiota

**DOI:** 10.3389/fmicb.2024.1272062

**Published:** 2024-03-01

**Authors:** Fan Wang, Lingmin Yu, Yuting Ren, Qianwen Zhang, Shanshan He, Minlei Zhao, Zhili He, Qi Gao, Jianguo Chen

**Affiliations:** ^1^Beijing YuGen Pharmaceutical Co., Ltd., Beijing, China; ^2^YingTan City people’s Hospital, Yingtan, China; ^3^Beijing Hotgen Biotechnology Inc., Beijing, China

**Keywords:** culturomics, breast milk, human milk microbiota, blood culture bottles, microbial repertoire

## Abstract

Viable microorganisms and a diverse microbial ecosystem found in human milk play a crucial role in promoting healthy immune system and shaping the microbial community in the infant’s gut. Culturomics is a method to obtain a comprehensive repertoire of human milk microbiota. However, culturomics is an onerous procedure, and needs expertise, making it difficult to be widely implemented. Currently, there is no efficient and feasible culturomics method specifically designed for human milk microbiota yet. Therefore, the aim of this study was to develop a more efficient and feasible culturomics method specifically designed for human milk microbiota. We obtained fresh samples of human milk from healthy Chinese mothers and conducted a 27-day enrichment process using blood culture bottles. Bacterial isolates were harvested at different time intervals and cultured on four different types of media. Using matrix-assisted laser desorption ionization time-of-flight mass spectrometry (MALDI-TOF MS) analysis, we identified a total of 6601 colonies and successfully obtained 865 strains, representing 4 phyla, 21 genera, and 54 species. By combining CBA and MRS media, we were able to cultivate over 94.4% of bacterial species with high diversity, including species-specific microorganisms. Prolonged pre-incubation in blood culture bottles significantly increased the number of bacterial species by about 33% and improved the isolation efficiency of beneficial bacteria with low abundance in human milk. After optimization, we reduced the pre-incubation time in blood culture bottles and selected optimal picking time-points (0, 3, and 6 days) at 37°C. By testing 6601 colonies using MALDI-TOF MS, we estimated that this new protocol could obtain more than 90% of bacterial species, reducing the workload by 57.0%. In conclusion, our new culturomics strategy, which involves the combination of CBA and MRS media, extended pre-incubation enrichment, and optimized picking time-points, is a feasible method for studying the human milk microbiota. This protocol significantly improves the efficiency of culturomics and allows for the establishment of a comprehensive repertoire of bacterial species and strains in human milk.

## Introduction

Breast milk is considered the best natural food for infants due to its complex nutritional and bioactive composition (Moubareck, 2021). The World Health Organization recommends exclusive breastfeeding for the first 6 months of a baby’s life to meet their nutritional needs (Bosi et al., 2016). While breast milk was once believed to be sterile, recent evidence suggests that it contains diverse microbial communities, primarily belonging to *Firmicutes*, *Proteobacteria*, and *Actinomycetota* (Marin-Gómez et al., 2020). Exclusively breastfed infants consume approximately 800 mL of breast milk per day, which contain 1 × 10^4^−1 × 10^7^ bacteria. Breast milk provides infants with a continuous supply of beneficial bacteria (Milani et al., 2017; Pannaraj et al., 2017), which play a role in the colonization and development of gut microbes, immune system maturation, cognitive development, and protection against diseases such as obesity, gastrointestinal disorders, and type 2 diabetes (Marseglia et al., 2015; Unar-Munguía et al., 2017; Ortega-García et al., 2018; Notarbartolo et al., 2022; Stinson and Geddes, 2022).

Advances in molecular technologies, particularly metagenomics, have revolutionized our understanding of microbial communities (Nayfach et al., 2021) and their impact on health and disease (Lagier et al., 2016). However, these new technologies are often plagued by inadequacies and biases, especially amplicon sequencing technologies, including high detection limits and DNA extraction errors that mask the true nature of bacterial diversity in humans and the environment (Hugon et al., 2015; Almeida et al., 2019; Chang et al., 2019). Even among bacterial strains of the same species, there are many biological phenotypic differences that cannot usually be distinguished by amplified sequences (Forster et al., 2019). Additionally, the analysis of metagenomic datasets heavily relies on high-quality reference databases (Quince et al., 2017), which can be complemented by traditional culture methods (Lagier et al., 2012).

Microbial culturomics is a highly effective method to obtain bacterial strains in a microbiota by isolating microorganisms on a large scale (Lagier et al., 2016, 2018), allowing for the identification of species with low abundance that are not detectable by metagenomics (Seck et al., 2019). A variety of effective culturomics techniques have been developed in the past few years to isolate gut bacteria. For example, one strategy involves using an LpxC enzyme inhibitor to prevent fast-growing bacteria from dominating the culture (Hou et al., 2019). Another approach is to use immunomagnetic beads to selectively isolate potential pathobionts for a particular disease of interest (Huang Z. et al., 2023). However, as our knowledge of microbiota continues to expand, traditional isolation strategies for discovering new microbes become more challenging. Therefore, there is a need for the development of more effective culturomics methods. Although culturomics is not sufficient to quantify the abundance of each species, it is the best method to obtain a live repertoire of the microbiota (Dickson, 2017). Therefore, to interpret the microbiota spectrum and decipher any microbial mechanism of action, it is essential to acquire a live microbial communities possible (Togo et al., 2019).

Culture-based microbiota methods offer two main advantages over sequencing technique. Firstly, they allow for the preservation of bacterial species from samples, which can then be used for further research such as genetic and phenotypic characterization of individual microbe (Lebeis, 2014; Almeida et al., 2019). Secondly, these methods are essential for investigating the impact of breast milk microbes on maternal and infant health at the strain level.

Previous studies have identified optimal culture conditions for different types of bacteria, including those found in the intestines and human fecal samples (Lagier et al., 2012, 2015). These studies have extended the pre-culture time, used specific nutrients (rumen fluid and sheep blood), and optimized the timing of sample collection to increase the successful rate of bacterial isolation. Chang et al. (2019) optimized the culturomics strategy in human fecal samples to pick more than 90% of bacterial species and reduce the workload by 40% by extending the pre-incubation time, supplementing with fresh medium and picking at optimal time-points. Prolonged incubation in blood culture bottles is a key step in the culturomics strategy used for human fecal samples (Lagier et al., 2012, 2015). However, there are no existing reports on using prolonged pre-incubation to isolate the human milk microbiota. During lactation, it was discovered that *Bifidobacterium lactis* Probio-M8 can transfer to breast milk through the entero-mammary route (Zhong et al., 2022). This suggests that it may be possible to cultivate microorganisms in milk using blood culture bottles for preincubation that mimic the intestinal environment.

The aim of this study was to develop a culturomics strategy specifically designed for the breast milk microbiota. In this research, we conducted a systematic comparison of bacterial species isolated from breast milk using various culture media. This comparison involved the incorporation of sheep blood in blood culture bottles, extending the pre-incubation time, and implementing sampling strategies at different time points. Ultimately, we successfully established a viable culturomics strategy to characterize the breast milk microbiome, consequently enhancing the effectiveness of culturomics and providing a robust foundation for future research.

## Materials and methods

### Samples collection

Breast milk samples were obtained from 9 healthy lactating mothers in Beijing who were 42 days postpartum. The volunteers were screened for various exclusion criteria including mastitis, infectious diseases (such as tuberculosis, viral hepatitis, and HIV), cardiovascular disease, metabolic disease (like diabetes), mental disorders, cancer or other serious diseases, and ongoing studies involving nutritional or pharmacological interventions. To collect the milk samples, the first 3 drops of foremilk were discarded, and the breast was cleaned with a sterile saline swab. Approximately 5 mL of milk was then collected by pump expression using sterile collection tubes with a volume of 15 mL. The samples were stored in a zip bag under anaerobic conditions at a temperature of 4°C after collection and transported to the laboratory within 2 h for testing. This study protocol was approved by the Ethics Committee of the Chinese Centre for Disease Control and Prevention (Beijing, China, agreement no. 2023-001) and all volunteers provided written informed consent prior to participation.

### Cultivation media

Columbia blood agar (CBA, OXOID, United Kingdom) is a universal medium for the cultivation of *Streptococci*, *Staphylococci* and other related bacteria (Schwab et al., 2019), while BHIS agar, brain-heart extract medium (BHI, OXOID, United Kingdom) supplemented with 0.01% hemin chloride (Sigma-Aldrich, United Kingdom) and 0.01% vitamin K1 (Source leaf organism, Beijing, China) for non-fastidious bacteria (Moossavi et al., 2021). The MRS agar, de Man, Logosa and Sharpe agar (MRS, Oxoid, United Kingdom) with 3% L-cysteine-HCl (Sigma-Aldrich, United Kingdom) for lactic acid bacteria (Moossavi et al., 2021) and TOS agar, Transgalactosylated oligosaccharides (TOS, Millipore, United Kingdom) with 0.04 g/mL mupirocin (Sangon Biotech, Shanghai, China) agar for *Bifidobacterium* (Margolles and Ruiz, 2021).

### Prolonged pre-incubation

Fresh breast milk was diluted 10-fold and 100 μL of each sample dilution was cultured on CBA, BHIS, MRS, and TOS agar plates. After 48−72 h of aerobic incubation at 37°C and anaerobic incubation (80% N_2_, 10% H_2_, and 10% CO_2_) at 37°C, colonies were identified by MALDI-TOF MS EXS2000 (Zybio Inc., Chongqing, China). The blood culture bottle consists of: peptone, beef paste, yeast powder, gelatine peptone, sodium chloride, glucose. 1 mL of each sample was also added to the blood culture bottles (Antobio, Zhengzhou, China) supplemented with 10% skim sheep blood (Baote Medical, Beijing, China) and incubated in an anaerobic incubator at 37°C (Ruskinn, United Kingdom). Samples were extracted from the pre-cultures at 6 time points on the 3rd, 6th, 9th, 15th, 21st, and 27th day post pre-incubation. The pre-incubation suspension was serially diluted 10-fold to 10^–10^-fold and 100 uL of each dilution was plated on CBA, BHIS, MRS, and TOS agar plates and incubated anaerobically at 37°C for 72 h. The colonies were then picked and identified by MALDI-TOF MS. After our initial identification, we would place the plates in anaerobic conditions to continue to observe if some slow-growing strains continued to grow.

### Colony picking strategies

Colony picking was performed according to the method of Chang et al. (2019) with some modifications. Plates containing 100−300 clones were selected for colonies picking using two methods. One method is called “experienced colony picking,” which means picking according to the size, color, and morphology of the colonies. With this method, 2−3 colonies with the same feature were picked. Another method is to pick all the colonies on the plate, which is called “picking all.” To reduce the workload, the 0 d samples were all selected by the “picking all” method, and other sample plates for the 3rd, 6th, 9th, 15th, 21st and 27th day post pre-incubation were carried out by “experienced picking.”

### MALDI-TOF MS identification

Colonies were identified using MALDI-TOF MS. Each deposit was covered with 1 μL matrix solution (saturated α-cyano-4-hydroxycinnamic acid in 50% acetonitrile and 2.5% trifluoroacetic acid) and dried at room temperature. MS data were analyzed using MDT Master (version 1.1). *E. coli* ATCC 25922 was used for mass calibration and optimization of instrument parameters to achieve a mean deviation in mass-to-charge ratio of less than 300 ppm after correction. An isolate was then labeled as correctly identified at the species level if at least one of the colony spectra had a score >2.0 and another of the colony spectra had a score >1.7. If the species could not be accurately identified by MALDI-TOF MS after three attempts, the isolate was identified by 16S rRNA gene sequencing analysis.

### Statistical analysis

Statistical analyses were performed using SPSS statistics 26.0. Significant differences at *P* < 0.05 between the means were calculated with One-way analysis of variance (ANOVA). GraphPad Prism (v.8) was used for graphing.

## Results

### Composition of culturable human milk microbiota under aerobic and anaerobic conditions

**FIGURE 1:**
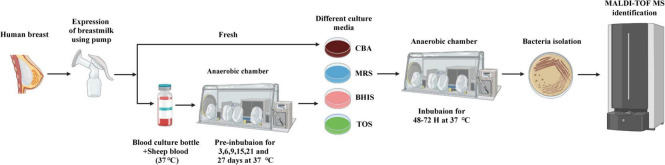
Experimental flow chart of culturomics for Human milk. Fresh milk samples were collected by pump expression and stored in a zip bag under anaerobic conditions. Direct inoculation: direct inoculation of the fresh milk sample was carried out on four different agar under aerobic and anaerobic conditions. Pre-incubation of blood culture bottles and inoculation on agar: The sample was pre-incubated in blood culture bottle supplemented with 5% sheep blood at 37°C, and extracted at different time-points (3, 6, 9, 15, 21, and 27 days) and cultured on four different media under anaerobic conditions. After 48–72 h of incubation in an anaerobic chamber, the bacterial colonies were sub-cultured. The identification of the colonies involved using MALDI-TOF mass spectrometry. In cases where the colonies could not be identified, they were further evaluated using 16S rRNA gene sequencing.

The experimental procedure can be seen in Figure 1. In summary, fresh milk samples were subjected to incubation under aerobic and anaerobic conditions. This included directly inoculating the samples onto four different agar plates. Additionally, pre-incubation was done in blood culture bottles containing 5% sheep blood under anaerobic conditions, and samples were taken at different time points. These samples were then sub-cultivated on four different types of media. The colonies that grew were identified using either MALDI-TOF or 16S rRNA gene sequencing. All identification results were analyzed from colonies picked within 72 h, and no new species were identified from colonies picked during the subsequent 48-hour incubation period. In this study, fresh breast milk samples obtained on day 0 served as control samples. The bacterial species isolated from pre-incubated samples were compared to those isolated from fresh breast milk.

**FIGURE 2:**
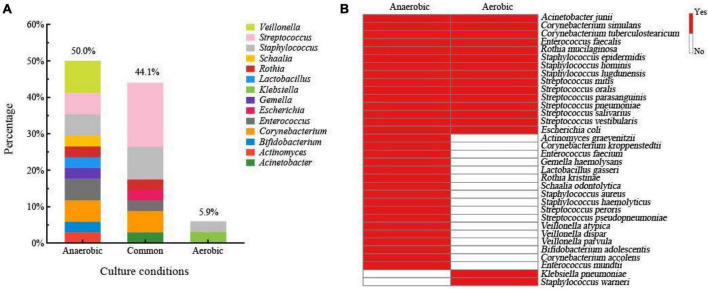
**(A)** Human milk microbiota composition of fresh breast milk samples at genus level isolated only from the anaerobic condition, only from the aerobic condition, and from both conditions. **(B)** Bacterial species composition in fresh breast milk samples grown only in anaerobic condition, only in the aerobic condition, and in both conditions.

Breast milk contains different types of bacteria that require different levels of oxygen to survive. The proportion of anaerobic bacteria in breast milk can provide insight into its overall quality (Jiménez et al., 2015; Patel et al., 2017; Togo et al., 2019). To study the bacterial composition of fresh breast milk, we conducted experiments using both aerobic and anaerobic conditions. We collected 3,588 bacterial colonies from four different culture media using a method that captured all the bacteria. We then identified the bacteria from nine fresh breast milk samples using MALDI-TOF MS. We found 14 different genera and 34 species of bacteria. Our analysis showed that 50% of the bacterial species were only found under anaerobic conditions, including notable examples such as *Lactobacillus gasseri, Bifidobacterium adolescentis, Veillonella atypica*, and other typical anaerobes. In contrast, 5.9% of the species were only detected under aerobic conditions (Figure 2A). We also identified 15 species that were present in both aerobic and anaerobic conditions, including *Staphylococcus epidermidis*, *Streptococcus salivarius*, and *Escherichia coli* (Figure 2B). Overall, 94.1% of the total bacterial species were isolated from fresh breast milk under anaerobic conditions. Based on our findings, we optimized the culturomics method for studying the human milk microbiota under anaerobic conditions to improve efficiency and reduce workload.

### An appropriate combination of culture media for human milk microbiota

**FIGURE 3:**
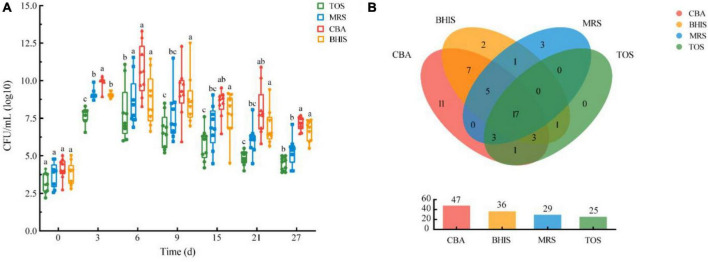
**(A)** Viable cell counts pre-incubated in blood culture bottles on four different media over time under anaerobic conditions. **(B)** Cultivable bacteria species numbers of human milk microbiota on different media under anaerobic conditions.

In this study, we examined different media for isolating breast milk microbes. We measured viable cell counts and bacterial species numbers to determine the most suitable media. We observed a significant increase in viable bacteria over a 27-day pre-incubation period compared to fresh breast milk, as shown in Figure 3A. CBA agar had the highest number of viable bacteria, followed by BHIS, and TOS agar had the lowest. The viable cell counts initially increased and then decreased, with CBA reaching a peak on the 6th day of pre-incubation. We isolated a total of 54 bacterial species from the four media. CBA had the highest percentage (87.0%), followed by BHIS and MRS (Figure 3B). We found four additional species, including specific *Lactobacillus* species, in MRS compared to TOS. No specific species were obtained from TOS. Overall, we isolated 51 species from CBA and MRS, accounting for 94.4% of all bacterial species in this study. The combination of CBA and MRS could be an optimized media for breast milk culturomics, capturing the microbial diversity of breast milk.

### An optimized picking time-points of pre-incubation in blood culture bottle

**FIGURE 4:**
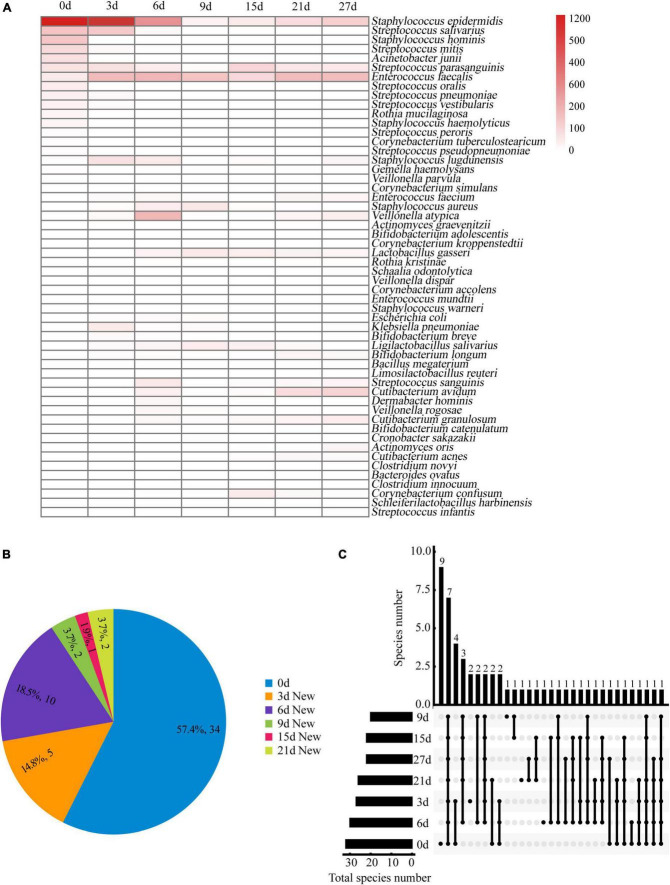
**(A)** Bacteria clones isolated from breast milk samples over time under anaerobic conditions. The bacteria identified at each time point are represented by red horizontal lines. The color scale at the right side of the figure indicates the number of bacterial clones isolated at each time point. **(B)** Distribution proportion and number of new bacterial species separated at each time point. **(C)** Upset plot showing how many species in different culture time were identified and their overlap. Horizontal bars show the total number of species in different culture time. Vertical bars show the number of same species at either one or more time-points, which are represented by multiple dots connected by a filled line.

A total of 6,601 colonies from 54 different species were identified at seven time points. Figure 4A shows the number of colonies and the distribution of bacterial species. On day 0, we isolated a maximum of 32 bacterial species. From days 3 to 27 (Figure 4B), we obtained 23 new species, indicating that the pre-incubation procedure significantly increased the likelihood of isolating additional bacterial species. The number of species increased most rapidly during the first 6 days of pre-incubation, with proportions of 57.4, 14.8, and 18.5% on days 0, 3, and 6, respectively. However, as time progressed, the rate of obtaining new species slowed down or even ceased. This may be due to the depletion of nutrients in the blood culture bottles and the accumulation of bacterial metabolites, which could not sustain the necessary nutrient requirements for bacterial growth. More than 90% of the bacteria species identified through the seven time-point sampling method could be isolated using the optimized combination of the three time-point strategy (0, 3, and 6 days).

Figure 4C shows eight species that were consistently found at all seven time points in the study. These include *Staphylococcus epidermidis*, *Staphylococcus lugdunensis, Streptococcus parasanguinis, Lactobacillus gasseri, Enterococcus faecalis, Enterococcus faecium, Veillonella atypica*, and *Klebsiella pneumoniae*. A total of 13 specific bacterial species were isolated from the samples taken at four time points (0, 3, 6, and 9 days), with the highest percentage (69.2%) found in fresh milk samples. On day 0, nine specific bacterial species were isolated, including *Bifidobacterium adolescentis, Actinomyces graevenitzii, Corynebacterium kroppenstedtii, Corynebacterium simulans, Corynebacterium accolens, Rothia kristinae, Staphylococcus haemolyticus, Staphylococcus hominis*, and *Enterococcus mundtii*.

Beneficial bacteria, such as *Lactobacillus, Bifidobacterium, Bacteroides*, and *Akkermansia muciniphila*, are typically present in breast milk in very small amounts (Togo et al., 2019; Lugli et al., 2020). While sequencing technology (Togo et al., 2019; Lugli et al., 2020) can identify these bacteria in breast milk, directly isolating them from fresh breast milk without pre-incubation enrichment is challenging. Our experiment yielded similar results, with *Lactobacillus gasseri* and *Bifidobacterium adolescentis* only being isolated from two fresh samples, without the presence of other typical infant-type species. However, through pre-incubation enrichment, we were able to successfully isolate *Bifidobacterium longum, Bifidobacterium breve, Bifidobacterium catenulatum, Ligilactobacillus salivarius, Limosilactobacillus reuteri, Bacteroides ovatus*, and other strains from additional samples. These results indicate that pre-incubation enrichment can improve the success rate of isolating beneficial bacteria in breast milk.

### Overview of the cultivable microbiota composition in breast milk

**FIGURE 5:**
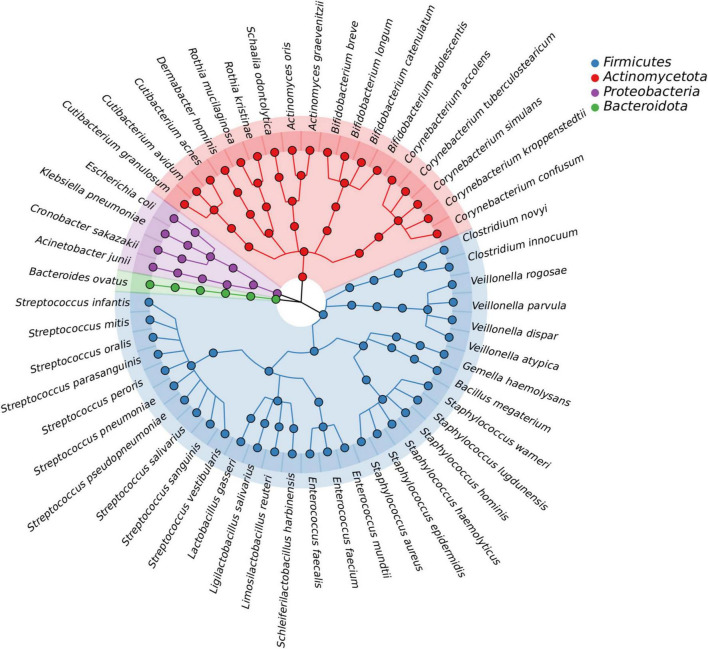
The bacteria species identified from all breast milk samples under anaerobic conditions. The outermost circle shows all 54 of the bacteria that were identified, with the four colors representing the phylum to which the bacteria belong. The evolutionary relationship between the bacterial species is shown by the lines in the middle.

**FIGURE 6:**
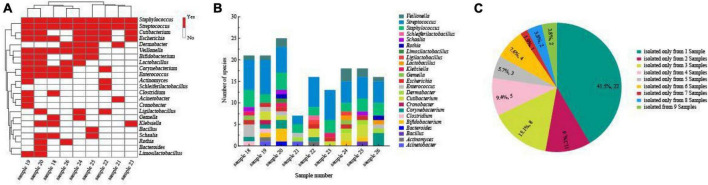
**(A)** Heatmap of the correlation between species numbers and breast milk samples under anaerobic conditions. The bacteria identified from each sample are represented by red horizontal lines. The color scale at the right side of the figure indicates the number of bacterial species isolated from each sample. **(B)** Human milk microbiota composition at genera level in each human milk sample. **(C)** Proportional distribution of bacterial species based on the separation source of breast milk samples.

In this study, we isolated a total of 865 strains from nine breast milk samples. These strains belonged to 4 phyla, 17 families, 23 genera, and 54 species. As shown in Figure 5, the majority of the bacteria were from the phyla *Firmicutes* (57.4%), *Actinomycetota* (33.3%), *Proteobacteria* (7.4%), and *Bacteroidota* (1.9%). *Firmicutes* mainly consisted of *Streptococcaceae* (32.3%) and *Staphylococcaceae* (19.4%), both of which were parthenogenetic anaerobes, followed by *Lactobacillaceae* (12.9%) and *Veillonellaceae* (12.9%). The second most abundant phylum, *Actinomycetota*, consisted of six families. The most frequently found genera in breast milk were *Streptococcus, Staphylococcus*, and *Escherichia*. The isolation method used in this study significantly increased the probability of detecting beneficial bacteria. The proportions of *Bifidobacterium, Ligilactobacillus*, and *Limosilactobacillus* increased to 44% (4/9), 33% (3/9), and 22% (2/9), respectively, (Figure 6A) surpassing the previously reported probabilities of isolating *Bifidobacterium* in breast milk samples, which ranged from 1.7 to 10.9% (González et al., 2013; Sakwinska et al., 2016). Sample 20 exhibited the highest diversity with 15 genera and 27 species, while sample 21 had the lowest diversity with only 7 genera (Figure 6B). Additionally, 41.5% of the bacterial species were found exclusively in a single milk sample, indicating a high variability of specific bacterial species in breast milk samples (Figure 6C).

## Discussion

Culturomics is a technology that combines various culture conditions with MALDI-TOF mass spectrometry and 16S rDNA sequencing to identify pure bacteria from complex ecosystems (Lagier et al., 2018). Pure bacterial cultures serve as a powerful tool in microbiome research, particularly for investigating the effects of host-microbe and microbe-microbe interactions. Although culturomics is widely applied to investigate microorganisms in the gut, vagina, mouth, and urinary tract (Wein et al., 2015; Fenollar and Raoult, 2016; Si et al., 2017; Chang et al., 2019), there have been relatively few studies on its application in human milk microbiota (Jost et al., 2013; Schwab et al., 2019; Treven et al., 2019). Therefore, there is a need to develop a culturomics strategy specifically for human milk samples. This would help establish a comprehensive microbial repertoire and uncover new insights. In this study, we propose a protocol to maximize the diversity of isolated human milk microbes and facilitate targeted selection of specific species.

Metagenomics and amplicon sequencing techniques uncover a broader range of bacterial diversity in fecal, vaginal, and breast milk samples compared to methods that rely on cultivation. However, cultivation-independent approaches may be ineffective for microorganisms that have not been previously cultured, isolated, and identified. Thus, we developed a culture-based strategy to analyze the breast milk microbiota under multiple conditions to capture viable microbial diversity through extensive isolation procedures. Traditional pure culture methods typically result in a limited number of species isolated from the breast milk microbiota. For example, Treven merely identified 25 different genera from 33 samples (Treven et al., 2019), whereas our study utilized a more efficient method and isolated 54 species from 23 genera in 9 samples. These findings indicate that improving culture methods can lead to a more comprehensive understanding of cultivable microorganisms in breast milk.

Previous studies have identified 820 types of bacteria and archaea in human milk, with the majority belonging to the *Proteobacteria* and *Firmicutes* phyla, specifically *Streptococcus* and *Staphylococcus* (Togo et al., 2019). Anaerobic microorganisms like *Bifidobacterium* and *Lactobacillus* are important for maternal and infant health (Lyons et al., 2020; Selma-Royo et al., 2021). However, these microorganisms were found in lower amounts in cases of mastitis and breast abscesses compared to healthy controls. Certain bacteria, such as *Bifidobacterium breve, Bifidobacterium bombi, Lactobacillus fermentum*, and *Akkermansia muciniphila*, were not found in mastitis and breast abscess samples (Togo et al., 2019). Recent studies have confirmed the importance of maternal probiotics for human health (Liu et al., 2020; Hou et al., 2023). *Bifidobacterium lactis* Probio-M8 can prevent respiratory infections in infants and improve symptoms of certain conditions like Alzheimer’s disease and Parkinson’s disease by regulating the gut microbiota (Cao et al., 2021; Mageswary et al., 2021; Sun et al., 2022). When lactating mothers were given Probio-M8, it was detected in both their milk and their infants’ fecal samples, indicating that it was transferred to the infant’s gut through breastfeeding (Zhong et al., 2022). *Lactobacillus fermentum* CECT5716 has also shown potential beneficial effects on inflammatory processes and the immune response, including mastitis and pediatric infections (Rodríguez-Sojo et al., 2021). In our study, we isolated a total of 6601 clones from fresh mature milk, mostly belonging to the *Firmicutes, Actinomycetota, Bacteroidota*, and *Proteobacteria*. Similar to previous studies, we found that *Staphylococcus* and *Streptococcus* were the most common genera in human milk, while *Bifidobacterium* and *Lactobacillus* were less abundant. We only found certain potential probiotic strains, like *Bifidobacterium longum, Bifidobacterium adolescentis*, and *Lactobacillus gasseri*, in a few milk samples. However, *Staphylococcus aureus, Streptococcus agalactiae*, and *Escherichia coli*, which can be both commensal and pathogenic, were the most common species found in all samples, regardless of the health status.

There are differences in the detection limits between high-throughput sequencing and culturomics methods, and it is commonly observed that sequencing and culturomics approaches complement each other rather than being inclusive in revealing microbial composition (Feehily et al., 2023). The objective of our study is to investigate cultureomics strategies specifically designed for cultivating microorganisms present in breast milk. The culturomics method isolates and identifies microorganisms based on their unique characteristics (Sakwinska and Bosco, 2019). However, it is important to note that this method is limited to microorganisms that can grow in specific culture media. In this study, we compared different culture media to find the best combination for isolating microorganisms in breast milk. We measured viable cell counts and bacterial species numbers to evaluate the cultivation characteristics of the human milk microbiota on the four different media. The results showed that the number of viable cells in breast milk samples varied among different populations. For example, breast milk samples from Slovenian and Swiss mothers had viable cell counts ranging from 1.2 to 5.5 log CFU/mL and log 2.6 to 6.1 CFU/mL, respectively, (Schwab et al., 2019; Treven et al., 2019). Our data from Chinese breast milk samples showed bacterial counts ranging from log 2.4 to 5.3 CFU/mL, which is consistent with previous studies. As the pre-incubation time increased, we observed that CBA had significantly higher live bacteria counts compared to the other three media. This suggests that CBA provided a more favorable environment for the growth of microorganisms that prefer its nutrients. The total number of bacteria reached its peak on the 6th day of incubation in CBA, indicating its suitability for capturing a wide range of microorganisms in breast milk. Furthermore, we found that *Lactobacillus gasseri, Limosilactobacillus reuteri*, and *Ligilactobacillus salivarius* were only detected on MRS medium, highlighting its superiority for isolating *Lactobacillus* species. The number of *Bifidobacterium* species isolated in CBA was comparable to that in TOS, suggesting that CBA could replace TOS for the isolation of *Bifidobacteria* in breast milk. In summary, our findings suggest that the combination of CBA and MRS media can effectively capture bacterial diversity and target potential probiotic species in breast milk. This makes it an ideal combination for culturomics studies.

In our study, we examined ways to improve the efficiency of isolating bacteria from human milk using culturomics. Culturomics is a meticulous and time-consuming task that requires careful consideration of efficiency and workload (Chang et al., 2019; Alou et al., 2020; Huang Y. et al., 2023). To address this challenge, we explored different picking methods, culture enrichment, and extended incubation time. Initially, we used the “picking all” method at the beginning and then switched to the “experienced picking” method. Previous research has indicated that the experienced picking method missed 32.9% of bacteria when compared to the picking all method at a single time point. However, as the pre-incubation time progressed, only 8.5% of the bacteria were missed (Chang et al., 2019). Hence, we believed that combining the experienced picking method with pre-incubation can compensate for the deviation between experienced picking and picking all. We also experimented with enriching blood culture bottles and extending the incubation time. This approach significantly increased the number of newly isolated species by 33%. We observed the highest count of viable cells on day 6 and were able to isolate 10 additional bacterial species, including potential probiotics like *Bifidobacterium longum*, *Bifidobacterium catenulatum*, *Ligilactobacillus salivarius*, and *Limosilactobacillus reuteri*. Compared to traditional methods, pre-incubation with blood culture bottles greatly enhanced the isolation efficiency of low-abundance bacterial species in breast milk. However, we found that prolonged incubation beyond day 6 was no longer beneficial for bacterial growth likely due to nutrient depletion in the culture medium. As a result, only 10% of new species were isolated in the later phase (7−27 days) using a time-consuming approach that yielded little success. To optimize the selection of time-points, we compared bacterial species isolated at seven different incubation times. Our findings revealed that 57.4, 14.8, and 18.5% of the total bacterial species were only isolated at day 0, 3, and 6, respectively. This indicates that 90.7% of the workload was completed within the first 43% of the total timeframe. By combining pre-incubation with blood culture bottles and selecting optimal time-points (0, 3, and 6 days), our new protocol enabled the isolation of more than 90.0% of the identified bacteria while reducing the workload by almost 57.0% compared to the original method. Therefore, our new protocol allows for the isolation of more new species from breast milk with the same amount of work effort.

## Conclusion

Culturomics is an effective way to study the microorganisms in human milk at the strain level. Our method combines culture and MALDI-TOF to isolate microorganisms from human milk. By using blood culture bottles, two culture media (CBA and MRS), and optimal time-points (0, 3, and 6 days), we can improve the efficiency of isolation and reduce the workload. However, there are limitations to our approach. We did not include control samples from the teat canal or skin, which could affect the accuracy of our findings. Using high-throughput sequencing methods in addition to culturomics would provide more comprehensive analysis. Further studies with more samples and access to high-throughput equipment are needed to develop improved methods.

## Data availability statement

The data presented in the study are deposited in the NCBI repository, with accession numbers PP325884, PP325885, PP325886, and PP325887. Further inquiries can be directed to the corresponding author/s.

## Ethics statement

This research was approved by the Ethics Committee of the Chinese Centre for Disease Control and Prevention (Beijing, China, approval number: 2023-001). All volunteers signed a written informed consent prior to participation.

## Author contributions

FW: Investigation, Methodology, Writing – original draft, Writing – review & editing. LY: Data curation, Methodology, Writing – original draft. YR: Data curation, Formal analysis, Investigation, Methodology, Writing – original draft. QZ: Formal analysis, Investigation, Methodology, Writing – original draft. SH: Data curation, Investigation, Methodology, Writing – original draft. MZ: Formal analysis, Investigation, Methodology, Writing – original draft. ZH: Writing – review & editing, Data curation, Investigation. QG: Project administration, Supervision, Writing – review & editing. JC: Supervision, Writing – review & editing.

## References

[B1] Almeida A. Mitchell A. L. Boland M. Forster S. C. Gloor G. B. Tarkowska A. (2019). A new genomic blueprint of the human gut microbiota. *Nature* 568 499–504. 10.1038/s41586-019-0965-1 30745586 PMC6784870

[B2] Alou M. T. Naud S. Khelaifia S. Bonnet M. Lagier J. C. Raoult D. (2020). State of the art in the culture of the human microbiota: New interests and strategies. *Clin. Microbiol. Rev.* 34 e00129–19. 10.1128/cmr.00129-19 33115723 PMC7605308

[B3] Bosi A. T. B. Eriksen K. G. Sobko T. Wijnhoven T. M. Breda J. (2016). Breastfeeding practices and policies in WHO European region member states. *Public Health Nutr.* 19 753–764. 10.1017/S1368980015001767 26096540 PMC4754616

[B4] Cao J. Amakye W. K. Qi C. Liu X. Ma J. Ren J. (2021). Bifidobacterium lactis probio-M8 regulates gut microbiota to alleviate Alzheimer’s disease in the APP/PS1 mouse model. *Eur. J. Nutr.* 60 3757–3769. 10.1007/s00394-021-02543-x 33796919

[B5] Chang Y. Hou F. Pan Z. Huang Z. Han L. Bin L. (2019). Optimization of culturomics strategy in human fecal samples. *Front. Microbiol.* 10:2891. 10.3389/fmicb.2019.02891 31921067 PMC6927924

[B6] Dickson I. (2017). Gut microbiota: Culturomics: Illuminating microbial dark matter. *Nat. Rev. Gastroenterol. Hepatol.* 14:3. 10.1038/nrgastro.2016.189 27876768

[B7] Feehily C. O’Neill I. Walsh C. Moore R. Killeen S. Geraghty A. (2023). Detailed mapping of Bifidobacterium strain transmission from mother to infant via a dual culture-based and metagenomic approach. *Nat. Commun.* 14:3015. 10.1038/s41467-023-38694-0 37230981 PMC10213049

[B8] Fenollar F. Raoult D. (2016). Does bacterial vaginosis result from fecal transplantation? *J. Infect. Dis.* 214:1784. 10.1093/infdis/jiw472 27703036

[B9] Forster S. C. Kumar N. Anonye B. O. Almeida A. Viciani E. Stares M. D. (2019). A human gut bacterial genome and culture collection for improved metagenomic analyses. *Nat. Biotechnol.* 37 186–192. 10.1038/s41587-018-0009-7 30718869 PMC6785715

[B10] González R. Mandomando I. Fumadó V. Sacoor C. Macete E. Alonso P. L. (2013). Breast milk and gut microbiota in African mothers and infants from an area of HIV prevalence. *PLoS One* 9:e92930. 10.1371/journal.pone.0092930 PMC3841168 24303004

[B11] Hou F. Chang Y. Huang Z. Han N. Bin L. Deng H. (2019). Application of LpxC enzyme inhibitor to inhibit some fast-growing bacteria in human gut bacterial culturomics. *BMC Microbiol*. 19:308. 10.1186/s12866-019-1681-6 31888576 PMC6937742

[B12] Hou F. Tang J. Liu Y. Tan Y. Wang Y. Zheng L. (2023). Safety evaluation and probiotic potency screening of Akkermansia muciniphila strains isolated from human feces and breast milk. *Microbiol. Spectr.* 11 e03361–22. 10.1128/spectrum.03361-22 36786638 PMC10103750

[B13] Huang Y. Sheth R. U. Zhao S. Cohen L. A. Dabaghi K. Moody T. (2023). High-throughput microbial culturomics using automation and machine learning. *Nat. Biotechnol.* 41 1424–1433. 10.1038/s41587-023-01674-2 36805559 PMC10567565

[B14] Huang Z. Chang Y. Hao K. Tan Y. Ding L. Wang L. (2023). Immunomagnetic−bead enriched culturomics (IMBEC) for isolating pathobionts from feces of colorectal cancer patients. *iMeta* 2:e100. 10.1002/imt2.100 PMC10989793 38868439

[B15] Hugon P. Dufour J. C. Colson P. Fournier P. E. Sallah K. Raolt D. (2015). A comprehensive repertoire of prokaryotic species identified in human beings. *Lancet Infect. Dis.* 15 1211–1219. 10.1016/s1473-3099(15)00293-5 26311042

[B16] Jiménez E. de Andrés J. Manrique M. Pareja-Tobes P. Tobes R. Martínez-Blanch J. F. (2015). Metagenomic analysis of milk of healthy and mastitis-suffering women. *J. Hum. Lact.* 31 406–415. 10.1177/0890334415585078 25948578

[B17] Jost T. Lacroix C. Braegger C. Chassard C. (2013). Assessment of bacterial diversity in breast milk using culture-dependent and culture-independent approaches. *Br. J. Nutr.* 110 1253–1262. 10.1017/S0007114513000597 23507238

[B18] Lagier J. C. Dubourg G. Million M. Cadoret F. Bilen M. Fenollar F. (2018). Culturing the human microbiota and culturomics. *Nat. Rev. Microbiol.* 16 540–550. 10.1038/s41579-018-0041-0 29937540

[B19] Lagier J. C. Hugon P. Khelaifia S. Fournier P. E. Scola B. L. Raoult D. (2015). The rebirth of culture in microbiology through the example of culturomics to study human gut microbiota. *Clin. Microbiol. Rev.* 28 237–264. 10.1128/cmr.00014-14 25567229 PMC4284300

[B20] Lagier J. C. Khelaifia S. Alou M. T. Ndongo S. Dione N. Hugon P. (2016). Culture of previously uncultured members of the human gut microbiota by culturomics. *Nat. Microbiol.* 1:16203. 10.1038/nmicrobiol.2016.203 27819657 PMC12094094

[B21] Lagier J. C. Million M. Hugon P. Armougom F. Raoult D. (2012). Human gut microbiota: Repertoire and variations. *Front. Cell Infect. Microbiol.* 2:136. 10.3389/fcimb.2012.00136 23130351 PMC3487222

[B22] Lebeis S. L. (2014). The potential for give and take in plant–microbiome relationships. *Front. Plant Sci.* 5:287. 10.3389/fpls.2014.00287 24999348 PMC4064451

[B23] Liu W. Chen M. Duo L. Wang J. Guo S. Sun H. (2020). Characterization of potentially probiotic lactic acid bacteria and bifidobacteria isolated from human colostrum. *J. Dairy Sci.* 103 4013–4025. 10.3168/jds.2019-17602 32113772

[B24] Lugli G. A. Duranti S. Milani C. Mancabelli C. Turroni F. Alessandri G. (2020). Investigating bifidobacteria and human milk oligosaccharide composition of lactating mothers. *FEMS Microbiol. Ecol.* 96 49–59. 10.1093/femsec/fiaa049 32188978

[B25] Lyons K. E. Ryan C. A. Dempsey E. M. Ross R. P. Stanton C. (2020). Breast milk, a source of beneficial microbes and associated benefits for infant health. *Nutrients* 12:1039. 10.3390/nu12041039 32283875 PMC7231147

[B26] Mageswary M. U. Ang X. Y. Lee B. K. Chung Y. L. F. Azhar S. N. A. Hamid I. J. A. (2021). Probiotic Bifidobacterium lactis Probio-M8 treated and prevented acute RTI, reduced antibiotic use and hospital stay in hospitalized young children: A randomized, double-blind, placebo-controlled study. *Eur. J. Nutr.* 61 1679–1691. 10.1007/s00394-021-02689-8 34825264 PMC8616720

[B27] Margolles A. Ruiz L. (2021). Methods for isolation and recovery of bifidobacteria. *Methods Mol. Biol.* 2278 1–12. 10.1007/978-1-0716-1274-3_1 33649943

[B28] Marin-Gómez W. Grande M. J. Pérez-Pulido R. Galvez A. Lucas R. (2020). Changes in the bacterial diversity of human milk during late lactation period (weeks 21 to 48). *Foods* 9:1184. 10.3390/foods9091184 32867028 PMC7554819

[B29] Marseglia L. Manti S. D’Angelo G. Cuppari C. Salpietro V. Filippelli M. (2015). Obesity and breastfeeding: The strength of association. *Women Birth.* 28 81–86. 10.1016/j.wombi.2014.12.007 25595034

[B30] Milani C. Duranti S. Bottacini F. Casey E. Turroni F. Mahony J. (2017). The first microbial colonizers of the human gut: Composition, activities, and health implications of the infant gut microbiota. *Microbiol. Mol. Biol. Rev.* 81 e00036–17. 10.1128/MMBR.00036-17 29118049 PMC5706746

[B31] Moossavi S. Fontes M. E. Rossi L. Fusch G. Surette M. G. Azad M. B. (2021). Capturing the diversity of the human milk microbiota through culture-enriched molecular profiling: A feasibility study. *FEMS Microbiol. Lett.* 368:fnab001. 10.1093/femsle/fnab001 33417698

[B32] Moubareck C. A. (2021). Milk microbiota and oligosaccharides: A glimpse into benefits, diversity, and correlations. *Nutrients* 13:1123. 10.3390/nu13041123 33805503 PMC8067037

[B33] Nayfach S. Roux S. Seshadri R. (2021). A genomic catalog of Earth’s micobiomes. *Nat. Biotechnol.* 39 499–509. 10.1038/s41587-020-0718-6 33169036 PMC8041624

[B34] Notarbartolo V. Giuffrè M. Montante C. Corsello G. Carta M. (2022). Composition of human breast milk microbiota and its role in children’s health. *Pediatr. Gastroenterol. Hepatol. Nutr.* 25 194–210. 10.5223/pghn.2022.25.3.194 35611376 PMC9110848

[B35] Ortega-García J. A. Kloosterman N. Alvarez L. Tobarra-Sánchez E. Cárceles-Álvarez A. Pastor-Valero R. (2018). Full breastfeeding and obesity in children: A prospective study from birth to 6 years. *Child Obes.* 14 327–337. 10.1089/chi.2017.0335 29912590 PMC6066191

[B36] Pannaraj P. S. Li F. Cerini C. Bender J. M. Yang S. Rollie A. (2017). Association between breast milk bacterial communities and establishment and development of the infant gut microbiome. *JAMA Pediatr.* 171 647–654. 10.1001/jamapediatrics.2017.0378 28492938 PMC5710346

[B37] Patel S. Vaidya Y. Patel R. Pandit R. Joshi C. Kunjadiya A. (2017). Culture independent assessment of human milk microbial community in lactational mastitis. *Sci. Rep.* 7:7804. 10.1038/s41598-017-08451-7 28798374 PMC5552812

[B38] Quince C. Walker A. W. Simpson J. T. Loman N. J. Segata N. (2017). Shotgun metagenomics, from sampling to analysis. *Nat. Biotechnol.* 35 833–844. 10.1038/nbt.3935 28898207

[B39] Rodríguez-Sojo M. J. Ruiz-Malagón A. J. Rodríguez-Cabezas M. E. Gálvez J. Rodríguez-Noga A. (2021). Limosilactobacillus fermentum CECT5716: Mechanisms and therapeutic insights. *Nutrients* 13:1016. 10.3390/nu13031016 33801082 PMC8003974

[B40] Sakwinska O. Bosco N. (2019). Host microbe interactions in the lactating mammary gland. *Front. Microbiol.* 10:1863. 10.3389/fmicb.2019.01863 31456777 PMC6701204

[B41] Sakwinska O. Moine D. Delley M. Combremont S. Rezzonico E. Descombes P. (2016). Microbiota in breast milk of Chinese lactating mothers. *PLoS One.* 11:e0160856. 10.1371/journal.pone.0160856 27529821 PMC4987007

[B42] Schwab C. Voney E. Garcia A. R. Vischer M. Lacroix C. (2019). Characterization of the cultivable microbiota in fresh and stored mature human breast milk. *Front. Microbiol.* 10:2666. 10.3389/fmicb.2019.02666 31824453 PMC6879428

[B43] Seck E. H. Senghor B. Merhej V. Bachar D. Cadoret F. Robert C. (2019). Salt in stools is associated with obesity, gut halophilic microbiota and Akkermansia muciniphila depletion in humans. *Int. J. Obes.* 43 862–871. 10.1038/s41366-018-0201-3 30206336

[B44] Selma-Royo M. Lerma J. C. Cortés-Macías E. Collado M. C. (2021). Human milk microbiome: From actual knowledge to future perspective. *Semin. Perinatol.* 45:151450. 10.1016/j.semperi.2021.151450 34274151

[B45] Si J. Lee C. Ko G. (2017). Oral microbiota: Microbial biomarkers of metabolic syndrome independent of host genetic factors. *Front. Cell Infect. Microbiol.* 7:516. 10.3389/fcimb.2017.00516 29326886 PMC5736563

[B46] Stinson L. S. Geddes D. T. (2022). Microbial metabolites: The next frontier in human milk. *Trends Microbiol.* 30 408–410. 10.1016/j.tim.2022.02.007 35282976

[B47] Sun H. Zhao F. Liu Y. Ma T. Jin H. Quan K. (2022). Probiotics synergized with conventional regimen in managing Parkinson’s disease. *NPJ Parkinsons Dis.* 8:62. 10.1038/s41531-022-00327-6 35610236 PMC9130297

[B48] Togo A. Dufour J. C. Lagier J. C. Dubourg G. Raoult D. Million M. (2019). Repertoire of human breast and milk microbiota: A systematic review. *Fut. Microbiol.* 14 623–641. 10.2217/fmb-2018-0317 31025880

[B49] Treven P. Mahnič A. Rupnik M. Golob M. Pirš T. Matijašić B. B. (2019). Evaluation of human milk microbiota by 16S rRNA gene next-generation sequencing (NGS) and cultivation/MALDI-TOF mass spectrometry identification. *Front. Microbiol.* 10:2612. 10.3389/fmicb.2019.02612 31803156 PMC6872673

[B50] Unar-Munguía M. Torres-Mejía G. Colchero M. A. Cosío T. G. (2017). Breastfeeding mode and risk of breast cancer: A dose-response meta-analysis. *J. Hum. Lact.* 33 422–434. 10.1177/0890334416683676 28196329

[B51] Wein S. A. Razvi H. Dave S. Reid G. Burton J. P. (2015). Re: The microbiome of the urinary tract–a role beyond infection. *J. Urol.* 194 1643–1644. 10.1016/j.juro.2015.09.053 26582671

[B52] Zhong Z. Tang H. Shen T. Ma X. Zhao F. Kwok L. Y. (2022). Bifidobacterium animalis subsp. lactis Probio-M8 undergoes host adaptive evolution by glcU mutation and translocates to the infant’s gut via oral-/entero-mammary routes through lactation. *Microbiome* 10:197. 10.1186/s40168-022-01398-6 36419187 PMC9682673

